# Genome-Wide Search for Host Association Factors during Ovine Progressive Pneumonia Virus Infection

**DOI:** 10.1371/journal.pone.0150344

**Published:** 2016-03-07

**Authors:** Jesse Thompson, Fangrui Ma, Meghan Quinn, Shi-Hua Xiang

**Affiliations:** 1 Nebraska Center for Virology, University of Nebraska-Lincoln, Lincoln, Nebraska, United States of America; 2 School of Veterinary Medicine and Biological Sciences, University of Nebraska-Lincoln, Lincoln, Nebraska, United States of America; 3 School of Biological Sciences, University of Nebraska-Lincoln, Lincoln, Nebraska, United States of America; University of Missouri, UNITED STATES

## Abstract

Ovine progressive pneumonia virus (OPPV) is an important virus that causes serious diseases in sheep and goats with a prevalence of 36% in the USA. Although OPPV was discovered more than half of a century ago, little is known about the infection and pathogenesis of this virus. In this report, we used RNA-seq technology to conduct a genome-wide probe for cellular factors that are associated with OPPV infection. A total of approximately 22,000 goat host genes were detected of which 657 were found to have been significantly up-regulated and 889 down-regulated at 12 hours post-infection. In addition to previously known restriction factors from other viral infections, a number of factors which may be specific for OPPV infection were uncovered. The data from this RNA-seq study will be helpful in our understanding of OPPV infection, and also for further study in the prevention and intervention of this viral disease.

## Introduction

Ovine progressive pneumonia virus (OPPV), or visna/maedi virus (VMV) in sheep [1–3], and caprine arthritis encephalitis virus (CAEV) in goats [4–6], all actually belong to one viral species called small ruminant lentiviruses (SRLV) according to current viral classifications [7]. These viruses are genetically very similar, with some OPPV strains being more closely related to CAEV than to other OPPV strains [7]. Additionally, the SRLV are able to infect across sheep and goat species [8–10]. OPPV infection in sheep has a prevalence of 36% in the USA [11]. OPPV infects sheep chronically, and can persist for the animal’s lifetime. OPPV infection usually causes multi-organ failure, and can lead to serious diseases such as pneumonia, mastitis, arthritis, wasting, and neurological disorders [7], bringing tremendous financial loss to the sheep industry, while seriously affecting animal health and well-being. OPPV displays a broad genetic diversity, similar to other lentiviruses such as human immunodeficiency virus type 1 (HIV-1) [12]. Subtype classification of SRLV has been also applied based on viral genetic sequence diversity [13–18], and recently circulating recombinant forms (CRFs) have also been identified in current epidemic transmission [19, 20].

Although OPPV was identified more than half a century ago, research on this virus has been limited [7]. Consequently, little is known about the viral infection process and pathogenesis, and there has not been an effective vaccine or treatment developed thus far. The traditional method for dealing with infected animals is to slaughter sick flocks in order to eliminate infected animals; however, this approach is slow, inefficient, and not economically sound. Therefore, a better solution is needed to prevent OPPV infection and maintain healthy flocks.

Infection by OPPV occurs naturally among adult animals (i.e., older than one year of age), via the respiratory route. However, infection of lambs through ingestion of infected colostrum and milk can also occur [21]. OPPV primarily targets macrophages and monocytes [22, 23], but not T-lymphocytes, of the infected hosts, and unlike HIV-1, it does not lead to CD4 T-cell depletion. Even though there is only limited sequence homology between the two viruses, OPPV resembles HIV-1 in a number of ways [12]. For instance, OPPV is also macrophage-tropic, causes slow disease progression, and infection is persistent, leading to life-long disease. Like HIV-1, OPPV belongs to the genus *Lentiviridae* in the family *Retroviridae* [11, 12], with a similar genomic organization, consisting of three major structural genes (*gag*, *pol* and *env*) and several accessory genes, such as *vif*, *tat* and *rev* [24]. The functions of these OPPV genes have not been well studied, and the molecular characterization of these genes is limited; therefore, it is not clear whether they function similarly to those of HIV-1.

The major goal of this research using the RNA-seq approach is to uncover the host cellular factors that are associated with OPPV infection. Identifying host factors associated with OPPV infection will help lead to therapeutic treatments. Further, this information can be used to develop strategies for preventing infection, such as the breeding of genetically resistant animals, and for the development of an effective vaccine, with the hope of virus eradication.

RNA-Seq is a recently developed, powerful approach for transcriptome profiling that uses the novel next-generation sequencing (NGS) technology [25, 26]. This approach has been fully evaluated, and offers the ability to quantify transcripts in the form of an entire transcriptome. It allows for the identification of up- or down-regulated genes on a genomic scale. RNA-Seq can also provide a more precise measurement of the levels of all gene transcripts and isoforms as compared to other existing methods such as Microarray [25, 27]. In this study, we used RNA-seq to probe the ovine host gene responses in OPPV infection.

## Materials and Methods

### Cell lines and plasmids

Permissive goat synovial membrane (GSM) cells and the OPPV viral strain (Dubois LMH19) was provided by Dr. Donald Knowles at Washington State University [28]. The ovine IFITM3 gene was synthesized from GenScript and cloned into the pcDNA3.1(+) vector, designed plasmid pIFITM3.

### OPPV infection and mRNA preparation

OPPV-permissive goat synovial membrane (GSM) cells were cultured in 6-well-plates in DMEM supplemented with 10% fetal bovine serum, with addition of 1% penicillin-streptomycin, and infected at an MOI of 5.0 when 70% confluent. Cells were collected at 0h, 12h, 24h, and 48h post-infection by scraping into TRIzol (Invitogen) reagent. Three independent infections or mock infections were harvested for each time point and pooled for sequencing. RNA extractions were performed according to manufacturer’s instructions. Following TRIzol extraction, RNA samples were subjected to further clean-up using RNeasy mini columns (Qiagen).

### Deep sequencing

The sequencing library of each RNA transcriptome sample was prepared with the TruSeq RNA Sample Preparation kit based on the protocol provided by the manufacturer (Illumina, San Diego, CA). The RNA samples from each group in equal amounts to generate one mixed sample per group. These mixed RNA samples were subsequently used to construct a complementary DNA (cDNA) library and perform Illumina deep sequencing. Briefly, a fragmentation buffer was mixed with magnetic beads and Oligo (dT) was used to isolate the messenger RNA (mRNA), and then the mRNA was fragmented into shorter fragments. The first strand of cDNA was then synthesized with random hexamer-primer using the mRNA fragments as templates. Double-stranded cDNAs were purified with the QiaQuick PCR extraction kit (Qiagen, Germany) and eluted with EB buffer for end repair and poly (A) addition. Finally, sequencing adapters were ligated to ends of the fragments, and the fragments were purified by agarose gel electrophoresis and enriched by PCR amplification to create a cDNA library. The sequencing was conducted in the UNL Genomic Research Core facility by an Illumina Solexa, and the Genome Analyzer System.

### RNA-Seq and pathway analysis

Genomic sequences and feature annotations of *Capra hircus* version 1.0 (Refseq assembly accession: GCF_000317765.1), and *Ovis aries* version 3.1 (RefSeq assembly accession: GCF_000298735.1) and *Visna/maedi* virus (NC_001452.1) were downloaded from NCBI website. Bowtie indices were generated using bowtie2-index program (bowtie version 2.1.0). Illumina sequence reads were mapped to reference genome sequence with TOPHAT 2.0.8. Transcripts and their isoforms were identified using CUFFLINKS 2.0.2. Differentially expressed genes were analyzed using CUFDIFF 2.0.2. The time series samples of 0, 12, 24, and 48 hours after infection and their corresponding uninfected samples were compared to find significantly up- and down-regulated genes. The heatmaps of the most significantly up- and down-regulated genes were generated with heatmap 2 R command.

Since the whole genomic sequence of OPPV viral strain (Dubois LMH19) was not available, we used a typical Iceland strain Visna/Maedi virus (VMV, NC_001452.1) for our viral dynamic analysis which was the highest homologous strain in the databases with the (81.26%) similarity in nucleotide sequences.

### Gene evolution and phylogenetic analysis

The completed genome sequences of *Capra hircus* and *Ovis aries* were used for all annotated genes mapping analysis with the all reads from the next-generation sequencing. Especially, due to the annotation coverage for the genome of the *Capra hircus*, the *Ovis aries* was used for the complimentary analysis. All individual gene or protein sequences were obtained from NCBI databases (details see below). The sequences were aligned using MUSCLE 3.8.31 [29] with output in PHYLIP interleave format. Phylogenetic tree was made using PHYML 3.0 with maximum likelihood algorithm [30]. The tree was visualized and edited using FigTree 1.4.0.

All protein sequences were downloaded from NCBI as follows: IFITM3 of *Ovis aries* (GI: 426252173), *Bos taurus* (GI:118151354), *Pan troglodytes* (GI:311771579), *Home sapiens* (GI:148612842), *Macaca mulatta* (GI:109104829), *Mus musculus* (GI:21539593), *Sus scrofa* (GI:319401913), and *Gallus gallus* (GI:50747606); ZAP of *Ovis aries* (GI:426228507), *Bos taurus* (GI:358411961), *Sus scrofa* (GI:294489384), *Tattus norvegicus* (GI:125630384), *Pan troglodytes* (GI:410212358), *Homo sapiens* (GI:27477136), *Macaca mulatta* (GI:383420071), and *Gallus gallus* (GI:61098418); APOBEC3F of *Ovis aries* (GI:199945618), *Bos taurus* (GI:118150804), *Sus scrofa* (GI:147905488), *Macaca nemestrina* (GI:315284485), *Pan troglodytes* (GI:410352161), *Homo sapiens* (GI:24416443), *Rattus norvegicus* (GI:74353701); Tetherin of *Ovis aries* (GI:295844819), *Bos taurus* (GI: 401664170), *Sus scrofa* (GI: 239916109), *Macaca mulatta* (GI: 259157110), *Pan troglodytes* (GI:298112972), *Homo sapiens* (GI: 4757876), *Mus musculus* (GI: 37674242); SAMHD1 of Ovis aries (GI: 426241450), *Bos taurus* (GI: 115496804), *Sus scrofa* (GI: 350594905), *Macaca mulata* (GI: 410442524), *Pan troglodytes* (GI: 387538824), *Homo sapiens* (GI: 38016914), *Mus musculus* (GI: 213418055), *Gallus gallus* (GI: 71895035), and *Danio reno* (GI: 229576924); MOV10 of *Ovis aries* (GI: 426216256), *Bos taurus* (GI: 115497510), *Sus scrofa* (GI: 350583523), *Macaca mulatta* (GI: 386781562), *Pan troglodytes* (GI: 410331601), *Homo sapiens* (GI: 14211540), *Mus musculus* (GI: 254540181), *Gallus gallus* (GI: 61098155), and *Salmo salar* (GI: 291190072).

### Plasmid transfection and viral infection

The IFITM3 plasmid and pcDNA3.1 (+) control vector were transfected into the GSM cells using Fugene-6 transfection reagent (Roche) in a 6-well plate. One day after the transfection, OPPV infection was performed using LMH19 viral stock at a higher MOI (5.0). The free viruses of medium samples were collected for RT assay one week after the viral infection.

### Reverse transcriptase (RT) assay

The reverse transcriptase assay was performed as standard DEAE filter paper (DE81, Whatman) assay method using a poly(A)dT12-18 template-primer for the radiolabeled thymidine 5’-triphospahte (TTP) incorporation into the synthesis cDNA molecule. The RT reaction samples were put on the DEAE paper followed by washing away unincorporated ^3^H-dTTP. The radioactive intensity retained on the DEAE paper was quantified by scintillation counter [31, 32].

## Results

### Host cellular gene responses during OPPV infection

**Fig 1:**
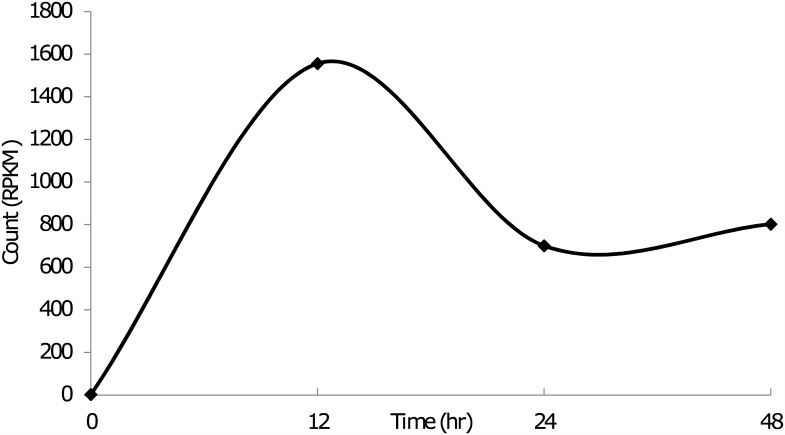
OPPV infection dynamics. Viral titers were measured at different time points post infection by measuring the reads RPKM (reads per kilobase per million). Viral strain tested, OPPV Dubois LMH19, permissive cells, goat synovial membrane cells (GSM).

During OPPV infection, the host responds to the infection and launches anti-viral defenses from both the innate and adaptive arms of the immune system. All of these responses are characterized by genetic regulation of many host cellular factors; some of these genes are up-regulated and some down-regulated, while others are kept consistent. These gene regulations can be monitored by changes in mRNA or protein expression levels. We used the newly developed RNA-seq technology for deep sequencing of the entire goat genome transcriptome expressed during OPPV infection. The whole goat genomic sequence was primarily used as the reference sequence for RNA-seq analysis [33]. The viral infection dynamics were monitored by measuring the reads of viral genes at 12, 24, and 48 hours (h) post-infection (p.i.). Our results indicate that OPPV infection dynamics peak at 12h p.i., and are reduced about one half by 24h p.i., remaining constant until 48h p.i. (Fig 1). It is assumed that after 24 h infection, the viral replication would reach an immunological balance with the host cells, it can maintain this level for a longer time in this *in vitro* tissue cultural system.

A total of approximately 22,000 goat host genes were detected and analyzed. In response to OPPV infection, these host genes were differentially expressed (DE) over time. If we use fold-change (FC) to measure the DE gene expression levels compared to those in the uninfected host cells at the same time points, the truly up- or down-regulated genes can be identified. Table 1 summarizes the results of the goat DE gene response to OPPV infection at different time points: 12h, 24h and 48h post-infection. This clearly shows that there are many more genes significantly differentially expressed (up or down) at the 12h and 24h time points, but largely reduced at 48h time point.

**Table 1:** Statistical summary of up- or down-regulated genes during OPPV infection.

DE gene type and category		No. of DE genes		
		12 hpi	24 hpi	48 hpi
Upregulated genes	1 < FC ≤ 1.5	444	660	55
	1.5 < FC ≤ 2	122	247	31
	FC > 2	91	163	92
	Total no. of up-regulated genes	657	1070	178
Downregulated genes	1 < FC ≤ 1.5	582	476	39
	1.5 < FC ≤ 2	197	158	12
	FC > 2	110	101	8
	Total no. of down-regulated genes	889	735	59

Benjamini-Hochberg adjusted p value (q value) less than 0.05

DE—differentially expressed genes

FC—log2 fold change compared with untreated sample at the same time point

**Fig 2:**
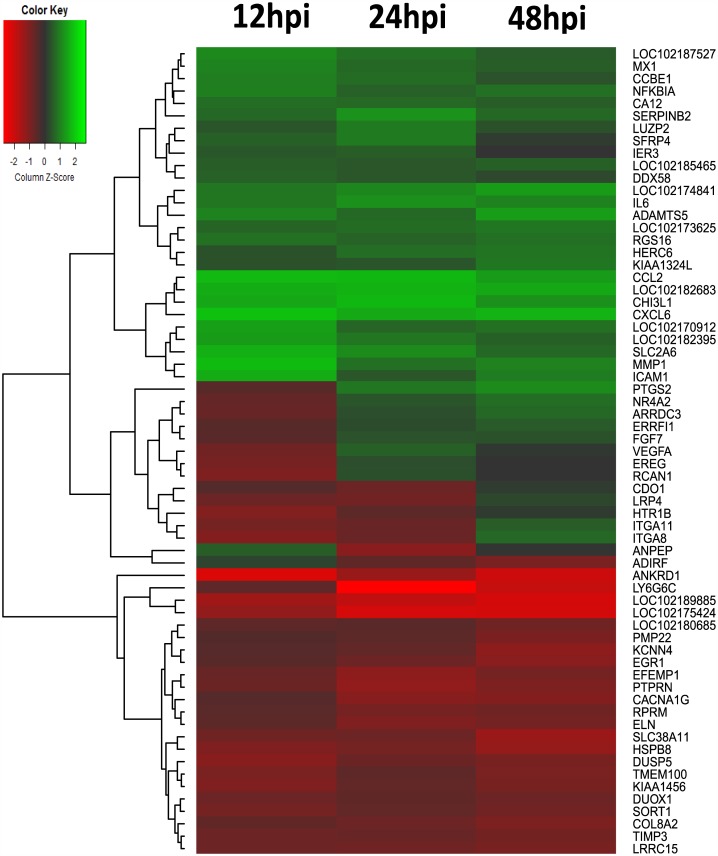
Heatmap of OPPV infection at different times post infection. Color green, indicates genes down regulation, color red, up-regulation. The color intensity from green to red indicates the gene expression levels. The expression profiles can be also clustered based on their similarities. The gene list of fold-changes during viral infection. Viral strain, OPPV Dubois LMH19, permissive cells, goat synovial membrane cells (GSM).

The profile of most significant cellular gene regulations were obtained from gene sequence analysis and data were shown as a heat-map (Fig 2) and a companion table (Table 2). The scores for their expression levels were calculated based on their fold-changes of infected samples and compared to the uninfected controls from the matching time point, as opposed to uninfected cells from time zero, so as to capture the transcriptional profiles in real-time. This allowed for fluctuations in gene expression which would occur as a result of time spent in culture even in the absence of viral infection to be removed from background in our dataset. The top 65 up- or down-regulated genes during OPPV infection at different time points were identified. From the heat-map, it was evident that some related genes were grouped and similarly differentially expressed (or up or down regulated) during OPPV infection (Fig 2). Some known cellular factors such as some cytokines and chemokines were identified (Table 2). However, in some cases, we found some most significant expressed genes were not identified through the *Capra hircus* genome sequence analysis but were identified when using *Ovis aries* genome sequence as a reference (Table 3). We have found out that this is due to the current genomic sequence annotation that contain some differences between goat and ovine. Actually, the gene sequences between goat and sheep are highly homologous and it is about 98.4% in average so that the data can be reasonable applied to *Capra hircus* when they are generated from *Ovis aries*.

**Table 2:** The most significant up- or down-regulated genes in *Capra hircus* during OPPV infection.

Gene	Encoding protein description	12hpi	24hpi	48hpi
CXCL6	Chemokine (C-X-C motif) ligand 6	4.06	3.88	2.47
MMP1	Matrix metalloproteinase-1	3.90	2.68	1.30
CCL2	The chemokine (C-C motif) ligand 2	3.83	3.27	2.73
SLC2A6	Solute carrier family 2, facilitated glucose transporter member 6	3.58	1.99	1.87
ICAM1	Intercellular Adhesion Molecule 1	3.51	2.55	0.82
CHI3L1	Chitinase-3-like protein 1	3.33	3.01	2.75
ADAMTS5	A disintegrin and metalloproteinase with thrombospondin motifs 5	2.37	3.34	1.18
MX1	Interferon-induced GTP-binding protein Mx1	2.37	1.76	1.17
NFKBIA	Nuclear Factor NF-Kappa-B P105 Subunit	2.28	2.21	1.04
CCBE1	Collagen and calcium-binding EGF domain-containing protein 1	2.23	1.50	1.26
IL6	Interleukin 6	2.01	2.70	1.95
RGS16	Regulator of G-protein signaling 16	1.85	2.20	1.08
CA12	Carbonic anhydrase 12	1.79	1.76	1.20
SERPINB2	Serpin Peptidase Inhibitor, Clade B (Ovalbumin), Member 2	1.60	2.05	2.00
DDX58	DEAD (Asp-Glu-Ala-Asp) Box Polypeptide 58	1.46	1.26	0.83
SFRP4	Secreted frizzled-related protein 4	1.43	0.95	1.56
ANPEP	Alanyl (Membrane) Aminopeptidase	1.33	0.80	-1.57
IER3	Immediate Early Response 3	1.20	0.71	0.97
LUZP2	Leucine zipper protein 2	1.10	1.46	1.57
KIAA1324L	KIAA1324-Like	1.02	2.39	0.80
HERC6	HECT And RLD Domain Containing E3 Ubiquitin Protein Ligase Family Member 6	0.99	2.31	1.28
VEGFA	Vascular endothelial growth factor A	-1.50	0.83	1.01
SORT1	Sortilin 1	-1.59	-0.84	-0.82
ITGA11	Integrin, Alpha 11	-1.61	1.75	-0.95
EREG	Epiregulin	-1.63	0.71	0.68
TMEM100	Transmembrane protein 100	-1.74	-1.04	-0.74
RCAN1	Regulator Of Calcineurin 1	-1.85	0.73	0.70
HSPB8	Heat shock protein beta-8	-1.88	-1.71	-1.17
KIAA1456	KIAA1456	-1.89	-0.98	-0.82
HTR1B	5-Hydroxytryptamine (Serotonin) Receptor 1B	-1.95	0.92	-0.69
ITGA8	Integrin alpha-8	-2.01	2.00	-0.96
DUSP5	Dual specificity protein phosphatase 5	-2.11	-1.01	-0.98
ANKRD1	Ankyrin Repeat Domain 1	-4.37	-3.04	-1.91

**Table 3:** Significant responsive genes identified through *Ovis aries* genomic sequence.

Gene	Description	Identity (%)	12h	24h	48h
SAA1	Serum amyloid A protein, lung cancer marker	97.917	10.09	5.54	5.84
MMP1	Matrix metalloproteinase-1	98.826	3.96	2.83	1.18
PTX3	Pentraxin3, antiviral activity	99.129	3.63	-	-
CHI3L1	Chitinase-3-like protein 1, biomarker of asthma	98.203	3.31	3.07	2.75
MX1	Interferon-induced GTP-binding protein Mx1	97.423	2.5	1.3	1.13
IFI44L	interferon-induced protein 44-like	98.637	2.11	0.81	0.55
LRRN3	Leucine-rich repeat neuronal protein 3	98.879	1.97	-	3.08
IFITM3	Interferon-induced transmembrane protein 3	98.148	1.32	-0.06	0.14

* Nucleotide sequence identity between the homolog genes in *Capra hircus* and *Ovis aries*

It is exciting that our RNA-seq data has captured a genome-wide picture of host gene expression during OPPV infection. Within the most up- or down-regulated genes are factors that are clearly related to OPPV infection (Tables 2 and 3). For instance, the SAA1 lung cancer biomarker, neuro-inflammation factor MMP1, the chemokine ligands (C-C motifs) CCL2, CCL5, and CCL20, and cytokines such as IL6, IL8, IL16, were all significantly modulated. Some known lentiviral restriction factors were affected, including tetherin (BST-2) and members of the IFITM family, along with the innate anti-viral factors OAS1 and OAS2.

**Fig 3:**
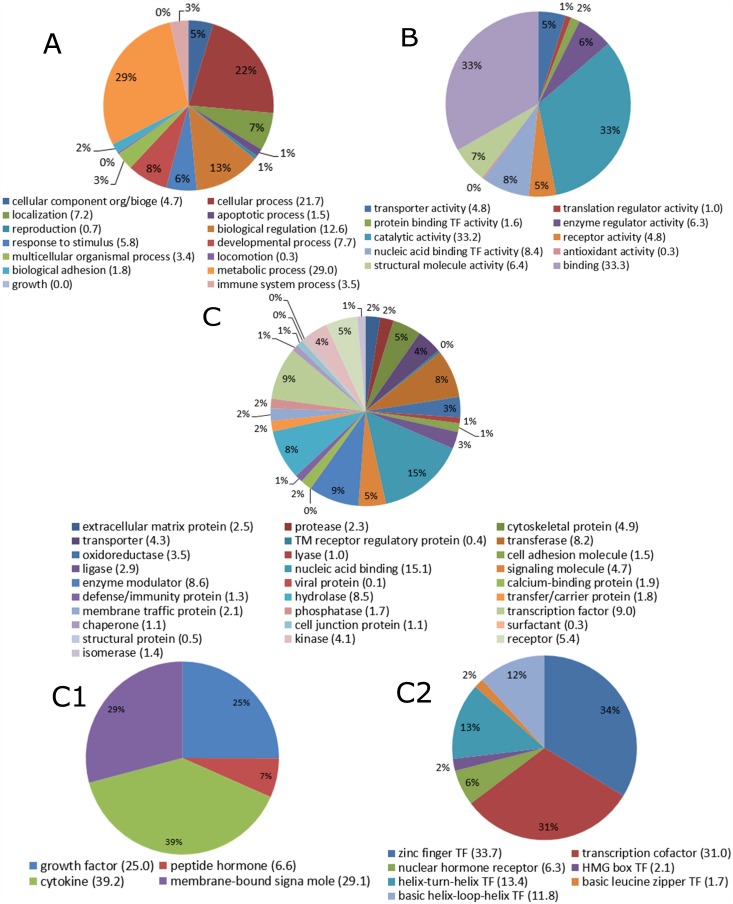
Differentially expressed genes in their functional pathways during OPPV infections. The responded genes from different biological processes (A), molecular functions (B), protein classes (C) were divided by different color areas with their percentiles of the pies. For the genes in protein classes which were further divided into signaling proteins (C1) and transcriptional factors (C2), and the different protein genes were also divided by different color areas with their percentiles of the pies.

When we performed the analysis of biological process or pathways, we found that a high degree of intra-cellular factors were most up-regulated, including metabolic and catalytic proteins, DNA and RNA binding proteins, transcriptional factors and signaling pathways factors (Fig 3). These analyses suggest that OPPV infection induces broad cellular responses which are involved in a series of biological pathway activations, such as signal transduction through interaction with cellular surface markers, leading to downstream intra-cellular events (Fig 3). The immune response pathways, such as chemokines and cytokines, transcription cofactors were also observed to be activated presumably as an antiviral defense, and were immediately up-regulated in response to the OPPV infection (Figs 2 and 3). It is interesting that the zinc finger transcription factors appear to be dominant in response to the viral infection (Fig 3C2).

### The host cellular factors that restrict viral infection, replication, and budding

**Fig 4:**
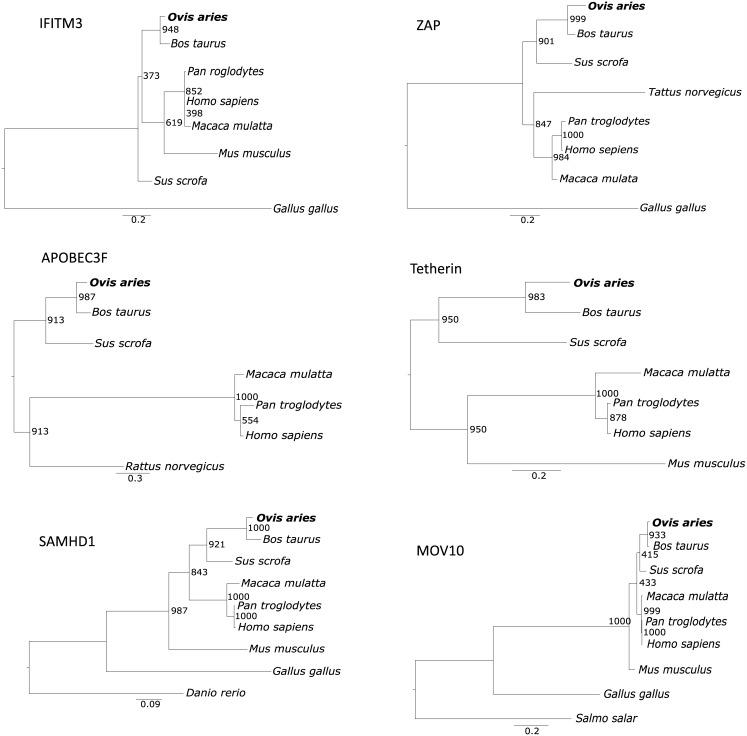
The evolutionary trees of six known restriction factors in human and other mammalian animal species. The protein sequences were used for analysis and building the trees. The program MUSCLE was used for sequence alignment and PHYML was used for building trees, and bootstrap 1000 steps were performed to reveal the branch knot possibilities. The unit of genetic distances is also shown in the diagrams. Ovine (*Ovis aries*) is in bold.

**Fig 5:**
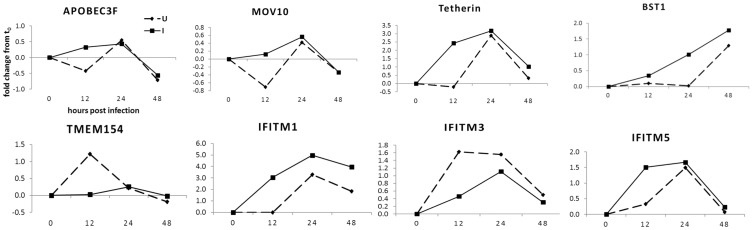
Differentially expressed profiles of known viral restriction factors during OPPV infection. Fold-changes (FC) determine by compared to the gene expressed levels in uninfected synovial membrane cells (GSM) at the matching control time points at 12, 24, and 48 hours post-infection.

Host restriction factors play important roles in retroviral infection and pathogenesis [34, 35]. There are many studies in human immunodeficiency virus (HIV), influenza and hepatitis viruses [34–40], but few studies in small ruminant lentiviruses (SRLV). In this study, our goal was to specifically search for cellular factors that can restrict OPPV infection because their identification of these will help to develop intervention strategies against viral infection and transmission. The phylogenetic relationships among some known lentiviral restriction factors of sheep (*Ovies aries*) and those of other mammals, including the closely related bovine (*Bos Taurus*), have been analyzed and shown in genetic evolution trees (Fig 4). It is suggested that the current known restriction factors could also exist in goats and ovine and have the same or similar function against OPPV infection. Our RNA-seq data identified a number of previously known host restriction factors against OPPV or other similar retroviruses such as HIV or SIV [34, 35, 38–42] which were significantly differentially regulated at 12h p.i. (Fig 5). They include members of the interferon-inducible transmembrane protein (IFITM) [43, 44], apolipoprotein B mRNA-editing catalytic polypeptide (APOBEC3) [45], interferon-inducible, transmembrane protein (Tetherin/BST-2 or BST-1) [46], and MOV10 [47–49] and TMEM154 (Transmembrane Protein 154) which has found to yield resistance to OPPV infection [28, 50]. Within these known restriction factors, some natural variants may have stronger capacities to withstand OPPV infection. Therefore, we will attempt to experimentally identify isotypes that show higher anti-OPPV activity, and isolate the natural genetic forms of sheep which could be utilized in a controlled breeding setting.

### The host cellular restriction factor IFITM3 against OPPV infection

**Fig 6:**
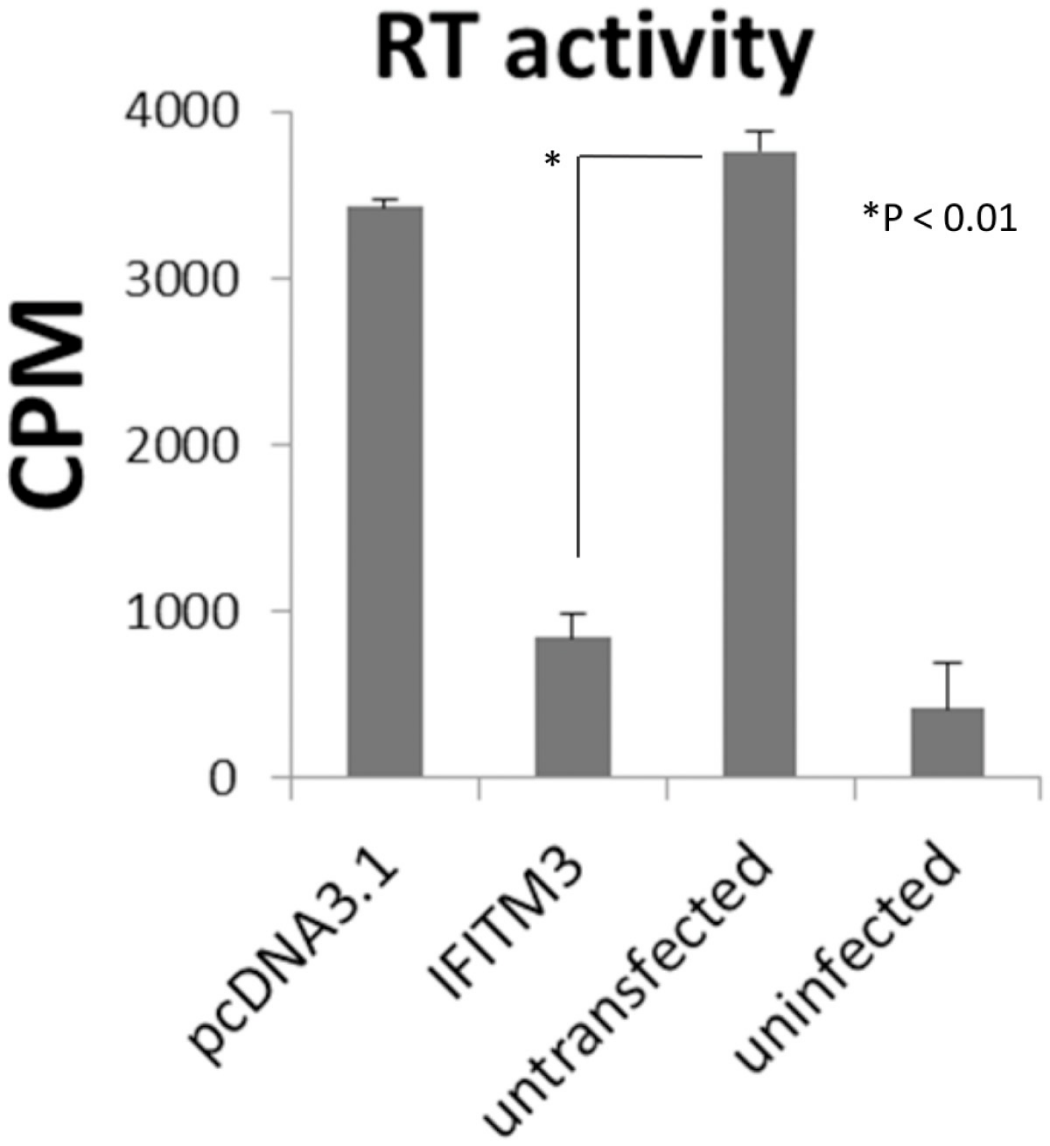
Effects on OPPV infection through over expression of IFITM3 gene in goat synovial membrane cells (GSM). Viral titers were measured by reverse transcriptase (RT) activity.

The objective of this study is to uncover the cellular factors that may be associated with the SRLV infection in a genomic scale. Therefore, in order to evaluate our RNA-seq data reliability in this genome-wide screening, we have chosen the cellular restriction factor IFITM3 for experimental testing. The sheep IFITM3 gene was synthesized and cloned into the pCDNA3.1 expression vector. The plasmid containing the sheep IFITM3 gene was transfected into the OPPV permissive cells, goat synovial membrane cells (GSM), followed by OPPV infection. Three days after infection, the media were collected for viral titers assay and the results were shown in the Fig 6. It was evident that the overexpression of the IFITM3 protein leads to lower viral titers than those observed in un-transfected GSM cells as measured by reverse transcriptase (RT) activity (Fig 6). This data suggests that the IFITM3 gene mediates anti-viral restriction activity against OPPV infection. This data also supports the integrity of our RNA-seq data, by way of demonstrating interactions between host cells and viruses.

## Discussion

Our RNA-seq analysis of OPPV infection has revealed many interesting host cellular factors that may be associated with the viral infection process. Some of these factors may directly play essential roles during OPPV infection, while others may have undiscovered or indirect involvement with OPPV infection and pathogenesis.

In this study, we have confirmed some known cellular restriction factors that are associated with interference against other lentiviruses (e.g. HIV/SIV), such as Trim5alpha, APOBEC, Tetherin. Actually, Trim5alpha has been reported to inhibit OPPV infection [51]. Given its route of transmission and tropism for alveolar macrophages, factors that have not been previously reported appear to be relevant to OPPV disease, such as lung cancer biomarker (SAA1), asthma biomarker (CHI3L1), and neuro-inflammation complement factor (CFB). The interferon induced factors such as ISG17, TIM family members, macrophage inflammatory protein 3 (MIP3A), monocyte chemotactic protein-1 (MCP-1), RANTES (CCL5), CD14, TNF receptor CD40, and some interleukins (IL3, IL4, IL6, IL8, and IL16) were all up-regulated. It is surprising that some GPCR protein family members such as RTP4 (chemosensory transport protein 4), and interferon-induced GTP-binding protein (Mx1) also experienced up-regulation during OPPV infection. However, some ion channel genes (calcium channel or potassium channel) appeared to be down-regulated; and growth factors (EGF, EGR, VGF etc.) were also down; and eventually the transcription factors such as the elongation factors were also reduced during the OPPV infection. These responses could be indicative of cellular shutdown to prevent further viral infection.

Our fundamental goal for studying OPPV is to identify naturally occurring genetic restriction factors against infection, allowing for sheep/goat breeding programs that would selectively produce virally-resistant flocks. The end result of such endeavors will be the gradual control and elimination of OPPV infection and transmission from animal herds. To meet this challenge, we intend to further characterize and analyze the individual factors identified by our RNA-seq research.

In conclusion, our study using RNA-seq technology to search for host cellular association factors during OPPV infection has captured useful information on relevant or important genetic factors that are involved in the infection and pathogenesis of this agriculturally relevant virus. This data has provided significant insights into OPPV research which may lead to reduction in overall prevalence, and eventual elimination from animal herds.
